# Variation among conventional cultivars could be used as a criterion for environmental safety assessment of *Bt* rice on nontarget arthropods

**DOI:** 10.1038/srep41918

**Published:** 2017-02-07

**Authors:** Fang Wang, Cong Dang, Xuefei Chang, Junce Tian, Zengbin Lu, Yang Chen, Gongyin Ye

**Affiliations:** 1 State Key Laboratory of Rice Biology, Ministry of Agriculture Key Laboratory of Agricultural Entomology, Institute of Insect Sciences, Zhejiang University, Hangzhou 310058, China; 2 Institute of Plant Protection and Microbiology, Zhejiang Academy of Agricultural Sciences, Hangzhou, 310021, China; 3 Institute of Plant Protection, Shandong Academy of Agricultural Science, Jinan 250100, China; 4 Institute of Virology and Biotechnology, Zhejiang Academy of Agricultural Sciences, Hangzhou, 310021, China

## Abstract

The current difficulty facing risk evaluations of *Bacillus thuringiensis (Bt*) crops on nontarget arthropods (NTAs) is the lack of criteria for determining what represents unacceptable risk. In this study, we investigated the biological parameters in the laboratory and field population abundance of *Nilaparvata lugens* (Hemiptera: Delphacidae) on two *Bt* rice lines and the non-*Bt* parent, together with 14 other conventional rice cultivars. Significant difference were found in nymphal duration and fecundity of *N. lugens* fed on *Bt* rice KMD2, as well as field population density on 12 October, compared with non-*Bt* parent. However, compared with the variation among conventional rice cultivars, the variation of each parameter between *Bt* rice and the non-*Bt* parent was much smaller, which can be easily seen from low-high bar graphs and also the coefficient of variation value (*C*.*V*). The variation among conventional cultivars is proposed to be used as a criterion for the safety assessment of *Bt* rice on NTAs, particularly when statistically significant differences in several parameters are found between *Bt* rice and its non-*Bt* parent. Coefficient of variation is suggested as a promising parameter for ecological risk judgement of IRGM rice on NTAs.

To meet the demand for food in the face of relatively limited arable land, China has devoted great efforts into developing genetically modified (GM) crops, especially insect-resistant GM (IRGM) rice lines. Cry proteins isolated from *Bacillus thuringiensis (Bt*) are the most widely used insecticidal proteins in IRGM rice. Since the first *Bt* rice plant was developed in 1989, over a dozen *Bt* rice lines with high resistance to lepidopteran target pests have been developed^1^,^2^,^3^,^4^,^5^,^6^. Two *Bt* rice lines, *Bt* Shanyou 63 and Huahui-1, received biosafety certificates in Hubei province in 2009, but neither has yet been approved for agricultural production. The issues related to the commercialization of GM crops include ecological risk, food safety, biosafety regulation, adoption by farmers and public acceptance. A relatively well-developed regulatory system for risk assessment and management of GM plants has been developed in China^7^. It was predicted that farmers would value the prospect of increased yields and the reduced use of pesticides and would readily adopt the production of *Bt* rice, based on experiences with *Bt* cotton and virus-resistant papaya^8^,^9^. The main factor slowing the pace of commercialization of GM rice in China is low public acceptance, which arises out of fear for human health and the environment^10^.

Food safety assessments of GM crops have been conducted investigating both their intended and unintended effects. Intended effect assessments have focused on measuring the thermal stability, digestibility, toxicity and allergenicity of introduced proteins as well as their metabolites. Unintended changes were assessed through compositional comparisons between transgenic and non-transformed parent plants following the principle of substantial equivalence^8^,^11^. The compositional equivalence between GM crops and their counterparts was confirmed over the course of 20 years of testing^12^. In the case of *Bt* rice, compositional comparison assessments suggested that *Bt* rice products are substantially equivalent to their non-transgenic counterparts^13^,^14^,^15^. Ninety-day rodent subchronic feeding studies with Cry proteins or whole foods have also been conducted, suggesting that *Bt* rice seeds are as safe for use as foods as their non-transgenic counterparts^16^,^17^,^18^,^19^,^20^,^21^,^22^,^23^. Concerns about the potential chronic effects of GM foods have arisen in recent years. To address these issues, long-term animal feeding test was conducted, although it was not considered to be scientifically beneficial or justified^24^. Certain differences were found in some haematology parameters, serum chemistry parameters and relative organ weights, but no adverse effect of *Bt* rice was recognised, as all of the differences were within the historical normal range^25^,^26^.

Since *Bt* rice lines were developed, numerous laboratory and field tests have been conducted on the potential risk of these lines on the environment, focusing on nontarget arthropods (NTAs), soil ecosystems and gene flow. The effect on NTAs has attracted much public attention, due to the fear of negative effects on natural enemies and useful animals^27^,^28^,^29^,^30^,^31^. The assessment of GM crops on NTAs typically starts with laboratory experiments under worst-case scenarios following a tiered framework conceptually similar to that used for conventional pesticides^32^,^33^. Most of these tier-1 studies have indicated that Cry proteins have no direct toxicity on NTAs^34^,^35^. However, recent dietary exposure tests have revealed adverse effects of Cry1C- or Cry2A-expressing *Bt* rice (T1C-19 and T2A-1) on *Propylea japonica* (Coleoptera: Coccinellidae), which was attributed to unintended changes in nutritional composition of *Bt* rice pollen rather than the toxicity of the expressed Cry proteins^36^. No significant effects of *Bt* rice lines T2A-1 or T1C-19 were found on biological parameters in laboratory or field abundance of the major pest, the brown planthopper (*Nilaparvatalugens,* Homptera: Delphacidae)^37^,^38^,^39^, and its main predator, *Cyrtorhinus lividipennis* (Hemiptera: Miridae)^40^. Nevertheless, a significantly higher survival rate was found in *Nephotettix cincticeps* (Hemiptera: Cicadellidae) fed on *Bt* rice T2A-1, while those fed on T1C-19 showed significantly longer nymphal duration and lower fecundity^41^. Similarly, *Bt* rice expressing Cry1Ab protein did not affect the fitness of *N. lugens* and its predators, *C. lividipennis, Ummeliata insecticeps* (Araneida: Linyphiidae) and *Pardosa pseudoannulata* (Araneida: Lycosidae), when nontarget pests were used as prey^42^,^43^,^44^. Meanwhile, negative effects of *Bt* rice expressing Cry1Ab protein on NTAs such as *Stenchaetothrips biformis, N. lugens* and *Anagrus nilaparvatae* were also reported^45^,^46^,^47^. The potential risk of IRGM crops on natural enemies has been debated in reviews and results differ primarily because of different analysis method with one method not accounting for prey quality^28^,^48^. Indications of the adverse effects of *Bt* crops on certain parameters of some soil organisms have also been reported. *Caenorhabditis elegans*, a bacteriophagous nematode, was negatively affected by both purified Cry1Ab protein and rhizosphere soil of *Bt*-maize expressing the Cry1Ab protein^49^. Significantly reduced reproduction was found in the springtail, *Folsomia candida* (Collembola: Isotomidae) when it was fed on *Bt* rice plant tissue^50^. However, neither positive nor negative effects have been determined conclusively as to whether it is harmful because significant difference is not necessarily equivalent to harm. And there is a lack of consensus on the criteria for environmental risk assessment, such as which types and levels of environmental changes are relevant and represent harm^51^.

Risk assessment characterises the likelihood and seriousness of a harmful effect. A definition of unacceptable harm is a prerequisite for environmental risk assessment. However, the policy protection goals set by the government are too broad and ambiguous to be directly applicable to risk assessment. In addition, operational harm criteria do not currently exist in most countries^52^. Most studies have adopted a comparative risk assessment approach in which the transgenic crop was only compared with the corresponding non-transgenic counterpart.

In the present study, we investigated the impact of different rice cultivars on a nontarget herbivore, *N. lugens*, together with two *Bt* rice lines under laboratory and field conditions, to determine if the variation between *Bt* rice and the non-*Bt* parent would exceed the range of variability among conventional rice cultivars. Biological parameters of *N. lugens*, including nymphal development duration, suvival rate, honeydew weight and fecundity under laboratory conditions, and also field abundance were used to estimate the variation range.

## Results

### Biological parameters of *N. lugens* on different rice cultivars in the laboratory

#### Nymphal development duration

**Figure 1:**
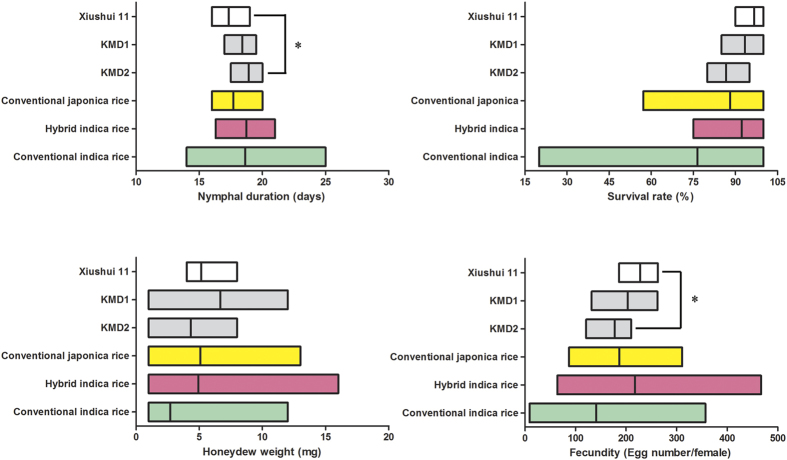
Biological parameters of *N. lugens* fed on different rice types. The biological parameters of *N. lugens* on two *Bt* rice lines KMD1/KMD2, and their non-*Bt* parental control Xiushui 11, as well as those on 14 conventional rice cultivars. The conventional rice cultivars were devided into three groups as conventional *japonica* rice, hybrid *indica* rice and conventional *indica* rice. In each low-high bar graph, the left border represents minimum value in the category, while the right border represents the maximum value; the line in the box represents the mean. Statistical difference was tested only between *Bt* lines and the non-*Bt* parent. *Indicates a significant difference according to one-factor ANOVA analysis and Tukey’s multiple-range test (*p* < 0.05).

The nymphal development duration of *N. lugens* fed on *Bt* rice lines was approximately 18.5 days, while on their non-*Bt* parent it was 17.3 days. When analysed independently, the nymphal duration of *N. lugens* was longer when fed on *Bt* rice versus on the non-*Bt* parent XS11, especially for insects fed on KMD2 (Fig. 1, F = 3.4135, df = 2,163, *p* = 0.0428). The coefficient of variation (*C*.*V*) among *Bt* rice lines and non-transgenic parent was 6.3%. The range of *N. lugens* nymphal duration among conventional *japonica* rice cultivars was 16.5 to 19.0 days, with a mean at 17.7 days and coefficient of variation at 7.1%; and the variation of nymphal duration on conventional *indica* rice cultivars was even larger (15.0 to 24.0 days, *C*.*V,* 18.5%; Table 1).

#### Survival rate

The survival rates of *N. lugens* nymphs fed on *Bt* rice lines and the non-*Bt* parent, together with 14 other rice cultivars, are shown in Fig. 1 and Table 1. The coefficient of variation in survival rates among *N. lugens* nymphs fed on *Bt* rice and non-*Bt* parent was very small (7.0%), compared with that of insects fed on conventional *japonica* rice cultivars (13.4%) and *indica* rice cultivars (32.8%). No statistically significant difference was detected in survival rate of *N. lugens* fed on *Bt* rice lines (both KMD1 and KMD2) from that of insects fed on the non-*Bt* parent XS11 (*F* = 2.333, df = 2,17, *p* = 0.1780). Only *N. lugens* fed on IR72 and IR42, which contain the *N. lugens* resistance genes *bph2* and *Bph3*, respectively, had significantly lower survival rates than all of the other treatments.

#### Honeydew weight

No significant difference in weight was found in honeydew produced by *N. lugens* female adults fed on *Bt* rice versus the non-*Bt* parent (*F* = 1.0943, df = 2,34, *p* = 0.3585). The *C*.*V* of honeydew among *Bt* rice and non-*Bt* parent was high (52.0%), but it was still lower than the *C*.*V* among different conventional rice types (60.3% for *japonica* rice and 107.1% for *indica* rice). The honeydew produced by *N. lugens* female adults fed on rice cultivars carrying resistance genes (IR26, IR72, IR42) was much lower than the others. However, statistical difference was only seen when females fed on the hybrid *indica* rice ZZY1 (Fig. 1 and Table 1).

#### Fecundity

The fecundity of *N. lugens* on KMD2 was significantly lower than that on XS11 when we compared *Bt* rice with the non-*Bt* parent independently (*F* = 3.6750, df = 2,44, *p* = 0.0405). However, mean levels of fecundity of females fed on the conventional *japonica* rice cultivars and hybrid *indica* rice cultivars were similar to those of insects fed on *Bt* rice lines (Fig. 1, Table 1). Meanwhile, the *C*.*V* of fecundity among *Bt* rice and non-*Bt* parent (21.3%) was smaller than that among different conventional rice types (31.9% for *japonica* rice and 64.8% for *indica* rice). And the fecundity of *N. lugens* on *Bt* rice KMD2 (177.3 eggs per female) fell within the 95% confidence interval of conventional *japonica* rice cultivars. Only the *N. lugens* females fed on hybrid rice cultivar LYPJ laid significantly more eggs than those fed on all other rice cultivars except TN1 and ZZY1. By contrast, significantly fewer eggs were laid by *N. lugens* fed on IR42 than on most of the other rice lines.

### Population density of *N. lugens* on different rice cultivars under field conditions

There was no significant difference in annual mean populations of *N. lugens* in the field among most of the cultivars, as shown in Table 2. No significant difference between *Bt* rice lines and the non-*Bt* parent was found when analysed independently either (*F* = 3.7641, df = 2, 8, *p* = 0.1204). The *C*.*V* of annual mean population between *Bt* and non-*Bt* parent was 21.8%, which was much smaller than that among conventional *japonica* or *indica* rice cultivars (58.4% and 67.6% respectively). Except for two rice lines, IR72 and ZJ22, on which the *N. lugens* population was consistently low throughout the experimental season, the population density varied significantly among sampling dates along with the development stages of *N. lugens* and rice (Table 2). Therefore, we further analysed the data from each sampling date. On 5 September, the *C*.*V* of *N. lugens* population density among *Bt* rice and its non-*Bt* parent (27.7%) was similar to that among conventional *japonica* cultivars (31.2%); but it was much smaller than those among conventional or hybrid *indica* rice cultivars. By contrast, on 24 September and 12 October, the *C*.*V* of *N. lugens* population density among hybrid *indica* rice was lower than that among conventional *indica* or *japonica* rice cultivars. Nevertheless, they were all higher than the *C*.*V* between *Bt* rice and non-*Bt* parent, although a significantly lower *N. lugens* population was detected on *Bt* rice KMD2 on 12 October when *Bt* rice lines were compared with the non-*Bt* parent XS11 independently (Supplementary Figure 1, *F* = 10.4630, df = 2,8, *p* = 0.0258).

### Principal component analysis (PCA)

**Figure 2:**
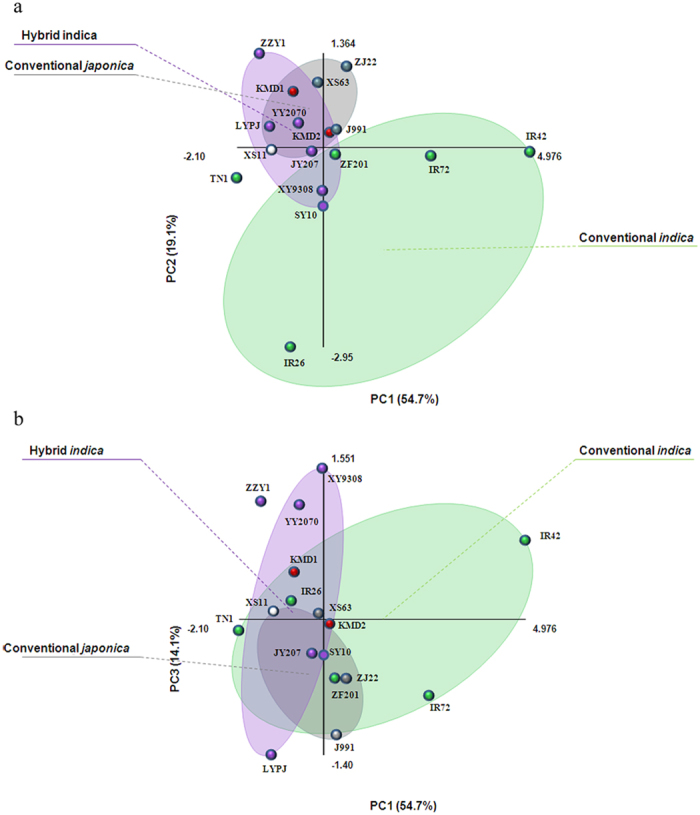
PCA score plots of *N. lugens* performance on different rice lines. Two-dimensional PCA score plots of the performance of *N. lugens* on different rice lines. The first three eigenvalues, which corresponded to approximately 87.9% of accumulated contribution, are shown in two separate plots. In each plot, the 15 conventional rice cultivars are divided into three groups: conventional *indica* rice (green circles), conventional *japonica* rice (grey circles) and hybrid *indica* rice (purple circles). Red circles show the two *Bt* rice lines and white circle represents the non-*Bt* parent. Factors are: nymphal development duration (X_1_), survival rate (X_2_), fecundity (X_3_), honeydew weight (X_4_), annual field population density (X_5_). PC1 = −0.4889X_1_ + 0.5498X_2_ + 0.4981X_3_ + 0.3418X_4_ + 0.3061X_5_ PC2 = 0.2235X_1_ − 0.0450X_2_ + 0.3047X_3_ + 0.5876X_4_ − 0.7141X_5_ PC3 = 0.3466X_1_ − 0.2257X_2_ − 0.2234X_3_ + 0.6679X_4_ + 0.5770X_5_.

We conducted PCA to identify the contribution rate of each parameter to the performance of *N. lugens* in the laboratory and in field (Fig. 2). The results showed the first three eigenvalues corresponded to ca 87.9% of the accumulated contribution. All 17 samples were represented two-dimensionally using their PC1, PC2 and PC3 scores in two separate plots. PC1 explained 54.7% of the variation and showed a separation of rice cultivars IR42 and IR72. The distribution of rice cultivars on PC1 was mainly determined by laboratory biological parameters, especially nymphal duration, survival rate and fecundity. PC2 accounted for 19.1% of the total variation and showed a separation of rice cultivars including IR26. The distribution of rice cultivars on PC2 was mostly affected by field population density. PC3 accounted for 14.1% of the variation. The weight of honeydew and field population density positively affected the distribution of rice cultivars on PC3. The two *Bt* rice lines did not show marked separation on PC1, PC2 and PC3 from their non-*Bt* parent XS11.

## Discussion

In the present study, the biological parameters and field abundance of *N. lugens* on *Bt* rice lines KMD1/KMD2 were compared with those on the non-*Bt* parent XS11 and 14 other conventional rice cultivars. Compared to variations in rice lines developed through modern biotechnology from their non-transgenic parent, variations in conventionally bred rice cultivars are even larger; however, they are usually accepted by the public without hesitation. We propose the use of variation in NTA biological parameters derived from a set of conventional cultivars with a history of safe production as a criterion for safety assessment of *Bt* rice on NTAs. When statistical differences were detected between *Bt* rice and the non-transgenic parent, the variation of the parameters in question were compared with the variation range of commercial rice lines which are considered to be normal for the crop. Then, the question of whether *Bt* rice is as safe as conventional rice can be answered. It is similar to that used for food and feed risk assessments^12^. The German Advisory Council on the Environment also defined harm as changes that go beyond the natural range of variability for a particular asset of value^51^.

The *Bt* rice line KMD2, which was reported to prolong the nymphal duration and affect the fecundity of *N. lugens*^45^, poses no harm when environmental safety is a protection goal. However, there have been reports of positive effects on nontarget pests or negative effects of *Bt* crops on nontarget arthropods. Mirid bug (Heteroptera: Miridae) population sizes increased in cotton and multiple crops were correlated with wide-scale adoption of *Bt* cotton in China^53^. *Bt*11 and Mon810 maize showed remarkable positive effects on the performance of the corn leaf aphid *Rhopalosiphum maidis* (Hemiptera: Aphididae)^54^. Increased survival rate of *N. cincticeps* on *Bt* rice T2A-1, and longer larval developmental time of the predator *P. japonica* have been reported when pollen of *Bt* rice T2A-1 and T1C-19 were used as food^36^,^41^. Parasitoid mummies are less abundant in *Bt* cotton CCRI 41 plots compared to conventional cotton plots^55^. For such situations, our proposal of using variation as a guideline would be helpful in safety judgment.

In the current study, low-high bar graphs demonstrate visually the range of each biological parameter of *N. lugens* on different rice types. Larger variations in range can be seen in conventional rice cultivars, especially in *indica* rice. Principle component analysis (PCA) explains the variance in the data through eigenvector-based multivariate analyses. By converting the observations of possibly correlated variables into a set of values of linearly uncorrelated variables, the 17 rice lines are visualised as a set of coordinates in two-dimensional pictures. From the two score plots, we can also see clearly that the variation between *Bt* rice and the non-*Bt* parent is small. As shown in Fig. 2, only the two resistant rice cultivars IR42 and IR72 are distinctly different from others. And only IR26 is separated from others due to high annual population density but low honeydew weight. Both low-high bar graphs and PCA score plots provide evidence that, compared with the non-transgenic parent, the resistant level of *Bt* rice to *N. lugens* was not altered by transgenic manipulation.

We calculated the 95% confidence intervals and coefficient of variation (*C*.*V*) of each parameter among different rice types to quantify the varation range. *C*.*V* is a standardized measure of dispersion of a probability distribution or frequency distribution. It shows the extent of variability in relation to the mean of the population, and is widely used as an index of reliability or variability in medical and biological sciences^56^. The confidence interval is also estimated based on the probability, but it is susceptible to sample size and distribution. Compared with the 95% confidence interval, the coefficient of variation might be a more promising parameter for such studies. In our case, the *C*.*V* between *Bt* rice and non-*Bt* parent was closer to that of conventional *japonica* rice or hybrid *indica* rice cultivars. Furthermore, most of the parameters investigated on *Bt* rice fell within the 95% confidence interval of conventional *japonica* rice or hybrid *indica* rice. Meanwhile, the background variability is high for field population densities especially among conventional indica rice cultivars, which was due to the inclusion of two *N. lugens* resistant rice lines (IR42, IR72). Although the field study was relatively small, due to logistical and regulatory constraints, the findings of the field study were supportive of the findings in laboratory study. As for most rice cultivars, the field population density represents both their attractiveness and tolerance. Inconsistent developmental stage of rice, sampling date and agronomic performance, such as greater tillers number, stronger stems and taller plants might all affect the results of field abundance investigation. So, it would be better to begin field investigation of *N. lugens* in late August with a sampling interval of 7–10 days. Rice cultivars with similar agronomic characters to the *Bt* rice evaluated should be used and a resistant and a sensitive comparator should also be set.

Traditionally cultivated crops with a history of safe use for consumers/domesticated animals have already been used as comparators in food and feed risk assessments according to the guideline of EFSA (2011)^57^. The current report presents the range of variation of different rice on NTAs. According to our observations, both laboratory and field experiment revealed a larger variation range in biological parameters and field abundance of *N. lugens* among conventional rice cultivars. Our results help confirm the notion that the natural variation range could be used as a criterion for environmental risk evaluation of GM crops on NTAs. When significant differences are found on NTAs between GM crops and comparators, the question of whether it is as safe as conventional rice could be answered by comparing the *C*.*V* from GM crops and their comparators with those among conventional rice cultivas, especially for situations where significant effects on nontarget arthropods of IRGM crops were found. Only those that fell outside the normal variation range should be suggested for further evaluation in terms of safety^12^. Further experiments are needed to develop well-established models for practical use in risk assessment of *Bt* rice on NTAs.

## Methods

### Experimental materials

Two *Bt* rice lines (KMD1 and KMD2) developed from two T_0_ plants expressing Cry1Ab driven by the maize ubiquitin promoter, as well as the untransformed parental cultivar Xiushui11 and 14 other non-transgenic rice cultivars, were used for laboratory and field evaluations. Both *Bt* rice lines had high resistance to stem borers and leaf folder under laboratory and field conditions^58^. The 14 non-GM rice cultivars included three conventional *japonica* rice cultivars, two conventional early season *indica* rice cultivars, three semilate *indica* rice cultivars with *N. lugens* resistance genes and six hybrid *indica* rice cultivars (Table 3).

### Insects

A colony of *N. lugens* was collected from the paddy field at the experimental farm of Zhejiang University, Hangzhou, Zhejiang Province, China, in 2011 and reared on susceptible ‘Taichung Native1’ (TN1) rice seedlings in nylon mesh cages in a phytotron (22 ± 2 °C, 60–70% relative humidity, and a 14:10 h light: dark regime.

### Laboratory experimental design

Rice seeds were soaked in deionised water at 25 °C for 2 days, germinated on a plastic board covered with plastic film at 35 °C for 1 day and grown in a controlled chamber at 25 ± 1 °C under a 14:10 h light: dark regime. The relative humidity was maintained at 85%. Ten-day-old seedlings were transplanted into plastic boxes and maintained in a greenhouse free from insect attack. For developmental duration and survival rate analysis, 30-d-old plants were transferred into glass tubes (38 × 250 mm) covered with nylon mesh, with one tube per seedling. The glass tube was filled with 5 cm (approximately 25 ml) Kimura B nutrient solution^39^. Ten newly hatched nymphs were infested onto each seedling. The plants were changed every five days until adult emergence. Six biological replicates were prepared per rice line. The survival rate and developmental duration of each nymph were recorded individually when all nymphs had emerged.

For reproduction analysis, the sex of each adult was determined on emergence. A newly emerged female and male from the same tested rice plant were mated and introduced onto a 60-d-old plant of the same type. Each plant was maintained individually in a plastic bottle (10 cm in diameter and 30 cm in height) covered with nylon mesh at the top for ventilation. Five to 15 biological replicates were performed per rice line. The plants were changed every 5 d until the adults died. The number of eggs laid per female was individually recorded with the aid of a stereomicroscope. Honeydew was also collected from individual female adults feeding on 60 d-old plants of the same type through the Parafilm sachets. Parafilm sachets were prepared as described by Heinrichs*et al*.^59^ and weighed. A previously starved 2-d-old female adult was transferred into each sachet, and then wrapped around the rice stem carefully. After feeding for 24 h, we removed the *N. lugens* and weighed the sachets containing the honeydew again. The difference between the two weights was the weight of honeydew^59^. Six to 12 biological replicates were prepared per rice line. All experiments were conducted in the same phytotron where the *N. lugens* were reared.

### Field Experimental Design

A total of 17 rice lines, including two *Bt* rice lines and their non-transgenic parent as well as 14 other conventional rice cultivars, were used for field studies at Changxing Agrotechnical Experiment Farm of Zhejiang University, Zhejiang, China in 2011. Rice seeds were sown on 1 July and transplanted on 30 July. The experiment was performed with a randomized block design with three blocks ×17 rice lines. Therefore, the experimental field was divided into 51 small plots. Each experimental plot was 2 × 1.5 m in size and separated on all sides by a 30-cm-wide walkway. Seedlings were hand transplanted at a rate of three seedlings per hill spaced 16 × 16 cm apart. The entire experimental field was surrounded by four border rows of TN1. Normal cultural practices for rice cultivation were followed during the entire experimental periods except that no insecticides were applied. The densities of *N. lugens* adults and nymphs were sampled by the beating tray method as described by Chen *et al*.^45^. On each sampling date, 5 hills were sampled at random along a diagonal line in each plot. Field population density was investigated every 20 days throughout the season beginning at the tillering stage.

### Statistical analysis

Data including nymphal survival rate, developmental duration, honeydew weight and fecundity were analysed by one-way ANOVA in a completely randomized design using the Data Processing System (DPS) package Version 15.10^60^, followed by Tukey’s multiple-range test. Population densities of *N. lugens* in the field were analysed using the GLM model repeated-measures analysis of variance in SAS v.9.1, where date was used as repeated factor (SAS Institute 2003)^61^. Field trail data was transformed by ln (x + 1). Tukey’s multiple-range test (α = 0.05) was used to identify the difference between cultivars in all the experiments. PCA was performed in Multibase 2013 in Microsoft Excel (www.numericaldynamics.com) by examining the correlation similarities between the observed measurements and four biological parameters and annual field population density of *N. lugens* were used as factors.

## Additional Information

**How to cite this article:** Wang, F. *et al*. Variation among conventional cultivars could be used as a criterion for environmental safety assessment of *Bt* rice on nontarget arthropods. *Sci. Rep.* **7**, 41918; doi: 10.1038/srep41918 (2017).

**Publisher's note:** Springer Nature remains neutral with regard to jurisdictional claims in published maps and institutional affiliations.

## Supplementary Material

Supplementary Figure

## Figures and Tables

**Table 1:** Biological parameters of *N. lugens* fed on different rice cultivars in the laboratory.

Rice types	Varieties	Nymphal duration (days)	Survival rate (%)	Honeydew weight (mg)	Fecundity (Eggs/female)
Conventional *japonica*	XS11	17.3 ± 1.2 (6)	96.7 ± 5.8 (6)	5.14 ± 1.35 (12)	227.6 ± 29.0(15)
	XS63	16.5 ± 0.5 (6)	75.7 ± 16.8(6)	6.50 ± 4.11 (8)	173.4 ± 74.1 (12)
	J991	18.0 ± 0.9 (6)	93.3 ± 5.8 (6)	2.75 ± 2.22 (12)	166.6 ± 61.8 (15)
	ZJ22	19.0 ± 0.9 (6)	86.7 ± 5.8 (6)	4.60 ± 2.88 (11)	177.4 ± 54.8 (15)
**Mean (*****C***.***V***)		**17.7 (7.1%)**	**88.1 (13.4%)**	**5.08 (60.3%)**	**186.6 (31.9%)**
95% confidence interval		17.2~18.2	80.6~95.6	3.79~6.38	166.1~207.1
Conventional *indica*	TN1	15.0 ± 0.9 (6)	96.7 ± 5.8 (6)	5.38 ± 4.24 (12)	240.6 ± 62.1 (15)
	ZF201	19.3 ± 1.7(6)	92.2 ± 2.5 (6)	2.67 ± 2.25 (12)	183.6 ± 83.9 (15)
	IR26	15.7 ± 1.0 (6)	92.6 ± 12.8 (6)	1.57 ± 0.79 (12)	126.3 ± 78.8 (15)
	IR72	19.2 ± 1.4 (6)	64.2 ± 12.4 (6)	1.33 ± 0.58 (8)	79.5 ± 54.7 (10)
	IR42	24.0 ± 0.9 (6)	36.7 ± 15.3 (6)	1.17 ± 0.41 (6)	36.2 ± 17.2 (5)
**Mean (*****C***.***V***)		**18.6 (18.5%)**	**76.5 (32.8%)**	**2.70 (107.1%)**	**140.9 (64.8%)**
95% confidence interval		17.3~19.9	67.1~85.8	1.62~3.78	111.4~170.5
Hybrid *indica*	ZZY1	18.1 ± 1.0 (6)	93.3 ± 5.8 (6)	9.17 ± 5.67 (12)	236.8 ± 110.7 (15)
	LYPJ	17.8 ± 0.9 (6)	93.3 ± 5.8 (6)	2.43 ± 3.36 (12)	343.3 ± 106.4 (15)
	JY207	18.7 ± 0.8 (6)	97.8 ± 3.9 (6)	3.60 ± 3.13 (12)	193.3 ± 44.7 (15)
	YY2070	19.6 ± 1.3 (6)	92.7 ± 6.4 (6)	6.71 ± 5.06 (12)	192.4 ± 52.6 (15)
	XY9308	20.0 ± 0.9 (6)	81.7 ± 7.6 (6)	5.25 ± 4.77 (9)	178.1 ± 64.2 (15)
	SY10	18.2 ± 1.4 (6)	94.5 ± 4.8 (6)	2.57 ± 2.30 (12)	155.8 ± 72.6 (15)
**Mean (*****C***.***V***)		**18.7 (6.9%)**	**92.2 (7.6%)**	**4.93 (93.9%)**	**217.1 (45.3%)**
95% confidence interval		18.3~19.2	89.8~94.6	3.44~6.40	190.0~244.2
***C***.***V*** **of** ***Bt*** **rice and non-*****Bt*** **parent**^a^		**6.3%**	**7.0%**	**52.0%**	**21.3%**
**Mean (*****C***.***V*****) of all conventional rice**		**18.4 (12.3%)**	**85.9 (20.4%)**	**4.25 (91.2%)**	**185.3 (49.8%)**
95% confidence interval		18.0~18.9	82.2~89.5	3.46~5.05	169.1~201.5

Data are represented as mean ± standard deviation (SD), numbers in brackets indicate sample size. *C*.*V*, coefficient of variation = (SD/Mean) × 100%.

^a^*C*.*V* of *Bt* rice and non-*Bt* parent represents coefficient of variation among two *Bt* rice KMD1, KMD2 and their parental control Xiushui 11, the values of which were shown in Fig. 1.

**Table 2:** Population density of *N. lugens* on different rice cultivars under field conditions.

Rice types	Varieties	5 September	24 September	12 October	Seasonal
Transgenic	KMD1	22.9 ± 10.1 a	16.5 ± 3.9 ab	239.1 ± 23.5 ab	92.8 ± 6.3 abc
	KMD2	26.1 ± 6.5 a	15.6 ± 5.5 ab	221.6 ± 14.5 ab	87.8 ± 5.5 abc
***C***.***V*** **of** ***Bt*** **rice and non-*****Bt*** **parent**		**27.7%**	**33.8%**	**22.6%**	**21.8%**
**Conventiional** ***japonica***	XS11	24.9 ± 5.8 a	18.4 ± 8.8 ab	331.6 ± 54.4 ab	120.5 ± 30.1 abc
	J991	14.2 ± 1.2 a	12.7 ± 10.2 ab	121.5 ± 74.3 abc	49.4 ± 23.0 bcd
	ZJ22	15.7 ± 4.7 a	19.5 ± 14.7 ab	64.1 ± 34.4 bc	33.1 ± 7.4 cd
	XS63	18.8 ± 4.7 a	20.9 ± 15.3 ab	147.0 ± 37.6 abc	62.2 ± 15.9 abcd
**Mean (*****C***.***V***)		**18.4 (31.2%)**	**17.9 (62.8%)**	**166.0 (68.6%)**	**66.3 (58.4%)**
95% confidence interval		14.7~22.0	10.7~25.0	98.7~238.4	41.7~90.9
**Conventional** ***indica***	TN1	92.1 ± 117.0 a	27.5 ± 11.5 ab	291.5 ± 24.9 ab	137.0 ± 30.9 abc
	ZF201	28.4 ± 6.6 a	25.5 ± 18.1 ab	388.4 ± 54.3 ab	84.7 ± 44.6 abc
	IR26	198.3 ± 85.4 a	22.3 ± 0.1 ab	376.6 ± 89.7 ab	203.9 ± 41.9 a
	IR72	42.3 ± 31.0 a	3.8 ± 2.9 b	74.2 ± 112.1 c	40.1 ± 46.3 d
	IR42	37.8 ± 29.8 a	15.5 ± 11.7 ab	128.5 ± 107.8 abc	60.6 ± 49.7 abcd
**Mean (*****C***.***V***)		**79.8 (108.9%)**	**18.9 (68.1%)**	**231.7 (64.6%)**	**105.3 (67.6%)**
95% confidence interval		31.7~127.9	11.8~26.0	141.3~322.2	65.9~144.7
**Hybrid** ***indica***	ZZY1	89.9 ± 108.5 a	25.3 ± 15.3 ab	202.3 ± 42.2 ab	105.8 ± 30.8 abcd
	LYPJ	89.4 ± 108.6 a	30.7 ± 11.8 a	150.7 ± 35.3 ab	90.3 ± 40.8 abc
	JY207	157.2 ± 118.1 a	16.5 ± 10.8 ab	115.2 ± 25.6 abc	96.3 ± 35.1 abc
	YY2070	30.9 ± 11.3 a	22.6 ± 14.5 ab	328.7 ± 49.5 ab	127.4 ± 15.7 abc
	XY9308	71.4 ± 56.1 a	16.1 ± 2.9 ab	414.8 ± 76.4 a	167.4 ± 27.8 ab
	SY10	100.5 ± 106.6 a	22.4 ± 6.4 ab	220.8 ± 68.4 ab	114.6 ± 46.9 abc
**Mean (*****C***.***V***)		**89.9 (97.2%)**	**22.3 (48.2%)**	**238.7 (48.1%)**	**117.0 (33.4%)**
95% confidence interval		46.4~133.3	16.9~27.6	181.6~295.9	97.6~136.4
**Mean (*****C***.***V*****) of all conventional rice**		**67.5 (117.4%)**	**20.0 (57.6%)**	**216.3 (58.8%)**	**99.6 (55.1%)**
95% confidence interval		43.7~91.3	16.5~23.4	177.2~255.4	83.1~116.0

Data are represented as mean ± SD (n = 3, No./rice hill for each date;for seasonal population density, n = 9). Values within the same sampling date followed by different lowercase letters differ significantly according to repeated-mesures ANOVA using GLM model and Tukey’s multiple-range test (*p* < 0.05). *C*.*V*, coefficient of variation = (SD/Mean) × 100%.

**Table 3:** Rice cultivars used for laboratory and field tests.

Rice varieties	Sub-species	Type	Season	BPH* resistance gene
Xiushui11(XS11)	*japonica*	Conventional	late	Non
KMD1	*japonica*	Transgenic *Bt*	late	Non
KMD2	*japonica*	Transgenic *Bt*	late	Non
Jia991(J991)	*japonica*	Conventional	late	Non
Zhejing22(ZJ22)	*japonica*	Conventional	late	Non
Xiushui63(XS63)	*japonica*	conventional	late	Non
TN1	*indica*	Conventional	early	Non
Zhefu201(ZF201)	*indica*	Conventional	early	Non
IR26	*indica*	Conventional	semilate	*Bph1*
IR72	*indica*	Conventional	semilate	*bph2*
IR42	*indica*	Conventional	semilate	*Bph3*
Zhongzheyou1(ZZY1)	*indica*	Hybrid	semilate	Non
Liangyoupeijiu(LYPJ)	*indica*	Hybrid	semilate	Non
Jinyou207(JY207)	*indica*	Hybrid	late	Non
IIyou2070(YY2070)	*indica*	Hybrid	late	Non
Xieyou9308(XY9308)	*indica*	Hybrid	late	Non
Shanyou10(SY10)	*indica*	Hybrid	late	*Bph1*

BPH*, brown planthopper, *N. lugens*.
