# Supplementary material for: Variation among conventional cultivars could be used as a criterion for environmental safety assessment of Bt rice on nontarget arthropods
Source: Sci Rep. 2017 Feb 7;7:41918. doi: 10.1038/srep41918 (PMC5294568; doi:10.1038/srep41918)

Variation among conventional cultivars could be used as a criterion for environmental safety assessment of *Bt* rice on nontarget arthropods

Fang Wang[1], Cong Dang[1], Xuefei Chang[1], Junce Tian[1,2], Zengbin Lu[1,3],Yang Chen[1,4], Gongyin Ye[1*]

Supplementary Figure 1 Field trial of *N. lugens* on different rice lines.

A, Low-high bar graphs of annual population densities on different rice types. The left border represents minimum value in the category, while the right border represents the maximum value; the line in the box represents the mean.

B, Population dynamics of *N. lugens* on *Bt* rice lines and their non-*Bt* parent. Error bars represent standard deviation. Different lowercase letters indicate significant difference within each sampling date according to one-factor ANOVA analysis and Tukey’s multiple-range test ($p < 0.05$).

Supplementary Figure1

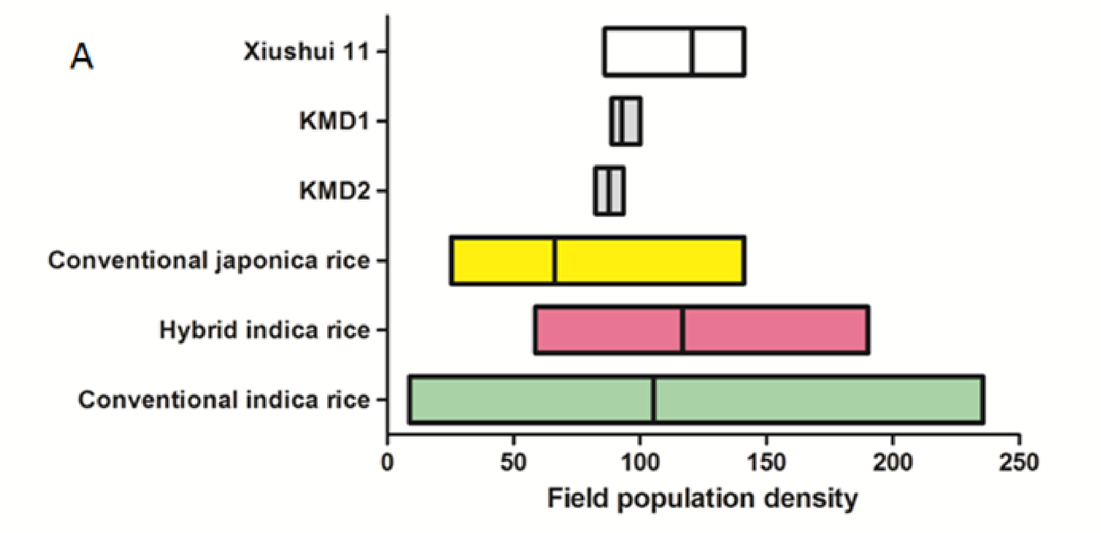

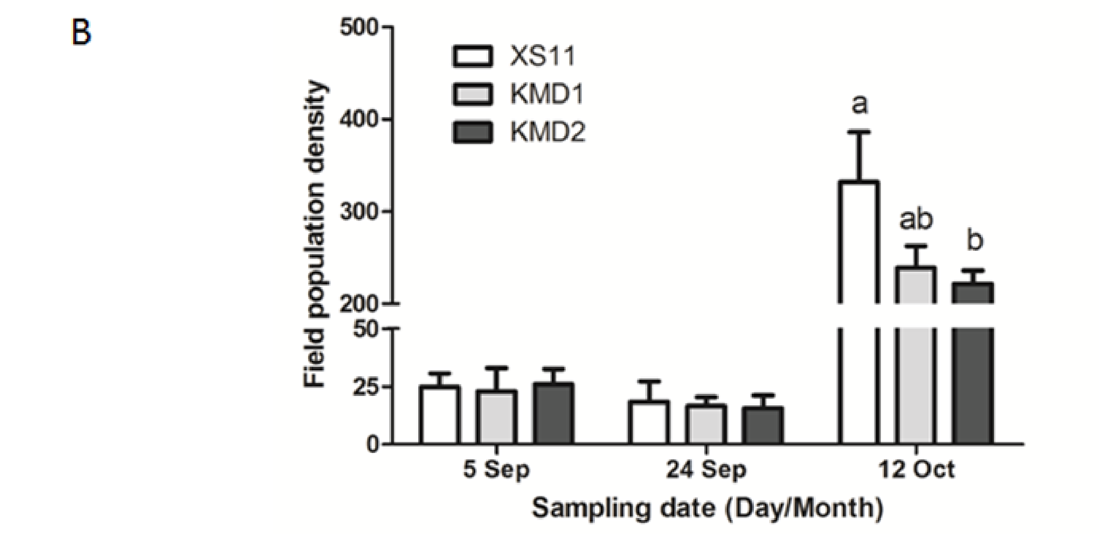

Supplement: Supplementary Figure [file srep41918-s1.pdf]
